# Neurosarcocystosis in Patient with HIV-Induced Immunodeficiency

**DOI:** 10.3201/eid3103.241361

**Published:** 2025-03

**Authors:** Tonje Skarpengland, Anders A. Tveita, Christopher F. Berntsen, Erik. E. Christensen, Magnhild E. Macpherson, Birgitte Stiksrud, Nils O. Hermansen, Pitt Niehusmann, Tine S. Oldereid, Espen Stjernstrøm, Hanne Brekke, Henrik V. Nielsen, Frank O.D. Pettersen

**Affiliations:** Oslo University Hospital, Ullevål, Oslo, Norway (T. Skarpengland, C.F. Berntsen, E.E. Christensen, M.E. Macpherson, B. Stiksrud, N.O. Hermansen, T.S. Oldereid, E. Stjernstrøm, H. Brekke, F.O.D. Pettersen); Institute of Clinical Medicine, University of Oslo, Oslo (A.A. Tveita); Oslo University Hospital, Rikshospitalet, Oslo (A.A. Tveita, P. Niehusmann); Statens Serum Institut, Copenhagen, Denmark (H.V. Nielsen)

**Keywords:** Sarcocystis, S. nesbitti, sarcocystosis, parasites, zoonoses, opportunistic infection, HIV, molecular diagnostic, Thailand, Norway

## Abstract

*Sarcocystis* is a genus of protozoan parasites that can infect various vertebrates. In humans, *Sarcocystis* infection usually is asymptomatic but might manifest as a mild gastroenteritis or extraintestinal myositis. We report a case of human central nervous system infection in Norway caused by *S. nesbitti* parasites.

The genus *Sarcocystis* consists of apicomplexan parasites, ≈200 species of which can infect reptiles, birds, and mammals; however, few species are zoonotic (*1*). Humans are definitive hosts of *S. hominis*, *S. suihominis*, and *S. heydorni*, shedding oocysts after ingestion of undercooked meat from intermediate hosts containing tissue cysts (*1*). Gastrointestinal infection is asymptomatic or causes a mild, self-limiting gastroenteritis (*2*). Human muscular sarcocystosis is a rare clinical syndrome associated with *S. nesbitti* infection mostly documented in Malaysia (*2*). The natural reservoirs of *S. nesbitti* parasites are probably reptiles, particularly snakes in Southeast Asia and Australia (*3*,*4*). Intermediate hosts, including humans, might develop tissue sarcocystosis after ingesting *S. nesbitti* sporocysts from fecally contaminated food or water. In Thailand, the prevalence of intestinal sarcocystosis is 7.0%–23.2% (*5*,*6*), but data regarding tissue sarcocystosis and *S. nesbitti* infection are scarce. We report a human case of *S. nesbitti* central nervous system infection in Norway.

The patient, a White male in his 70s, had lived in Norway for ≈40 years and visited Thailand for several months a year for 10 years. While in southern Thailand, he experienced increasing back pain and acute diplopia, aphasia, unilateral hemiparesis, and urinary and fecal incontinence. Imaging conducted in a clinic in Thailand revealed multiple brain lesions, and he returned to Norway for further investigations.

Upon the patient’s hospital admission in Norway, initial laboratory workup revealed an undiagnosed HIV infection (viral load 50,000 copies/mL, CD4+ T-cell count 116 cells/mm^3^). Magnetic resonance imaging showed numerous cortical and subcortical contrast-enhancing lesions in both cerebral hemispheres, along with multiple cerebellar, cervical, and thoracic spinal cord lesions (Figure). We noted hemorrhagic components and substantial perilesional edema (Figure). 18F-fluorodeoxyglucose (FDG) positron emission tomography–computed tomography demonstrated intense focal FDG uptake corresponding to areas of contrast enhancement found on magnetic resonance imaging. Apart from a diffusely increased signal in gluteal muscles, we noted no abnormal FDG uptake outside the central nervous system (CNS). The overall assessment suggested metastatic cancer, with opportunistic infection as a differential diagnosis.

**Figure:**
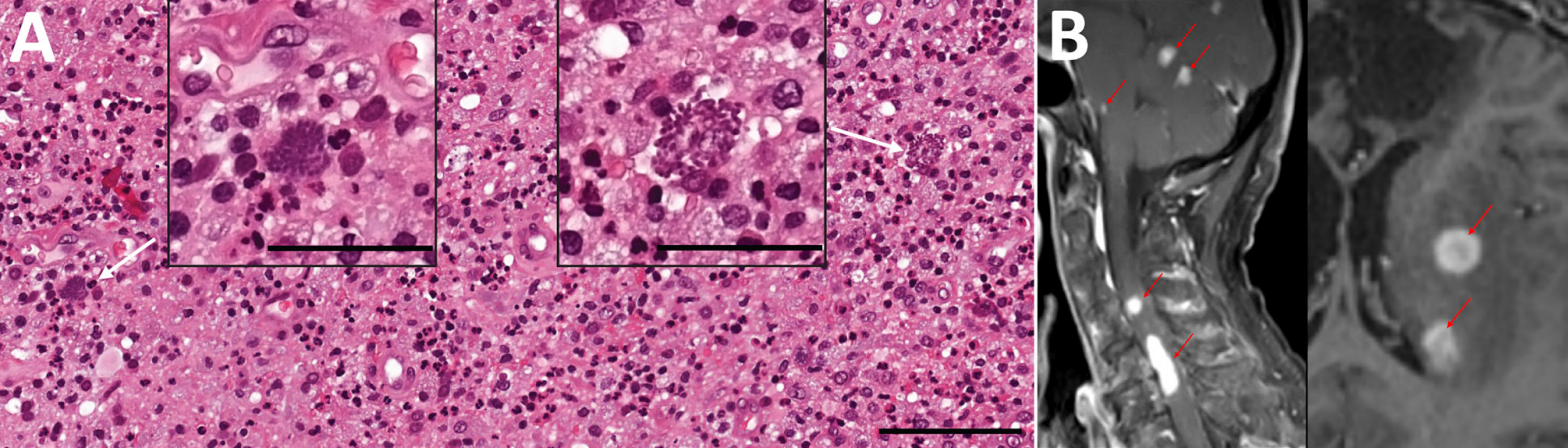
Microscopic findings and imaging results for patient with HIV-induced immunodeficiency preceding diagnosis of neurosarcocystosis, Norway. A) Light microscopic findings of structures resembling *Toxoplasma gondii* bradozoites (white arrows) in brain biopsy. Hematoxylin and eosin stain. Scale bars of enlarged images indicate 50 μm; scale bar of background image indicates 100 μm. B) Magnetic resonance imaging of cerebral and spinal cord lesions (contrast enhanced sagtittal T1 sequence, left panel, red arrows) and cerebral lesions with slight peripheral ring enhancement (contrast enhanced transversal T1 sequence, right panel, red arrows).

Cerebrospinal fluid (CSF) analysis showed an unremarkable leucocyte count (<4 × 10^6^ cells/L), but increased protein level (1.8 g/L) and albumin and IgG indices. Serologic test results were positive for *Toxoplasma gondii* IgG and negative for IgM. Blood and CSF were negative for *T. gondii* DNA. We detected asymptomatic reactivation of Epstein-Barr virus (EBV) and cytomegalovirus in blood. Results of additional microbiologic diagnostic analyses of other viruses, bacteria, fungi, and parasites (e.g., tuberculosis interferon-γ release assay and serologic and molecular testing of blood and CSF) were negative.

Histological examination of brain tissue revealed no signs of malignancy but indicated lymphohistiocytic infiltrates and singular structures resembling *T. gondii* bradyzoites (Figure). However, *T. gondii* immunohistochemical testing was inconclusive, and results for 2 different *T. gondii*–specific PCR assays were negative. Brain tissue PCR results were negative for herpes simplex virus 1 and 2, varicella zoster virus, cytomegalovirus, JC virus, 16S rDNA, internal transcribed spacer 2, and D1D2 fungal DNA. EBV PCR results were positive, but in situ hybridization displayed EBV-positive cells in a minute proportion of infiltrating lymphocytes, compatible with unspecific reactivity. We sent brain tissue and CSF to Statens Serum Institut (Copenhagen, Denmark) for metabarcoding analyses based on 16S and 18S DNA PCR combined with next-generation sequencing (*7*).

Faced with the presence of multiple space-occupying CNS lesions and evolving neurologic symptoms in the patient, we initiated treatment with dexamethasone pending further diagnostic workup. We started the patient on antiretroviral therapy, and after histologic assessment of the brain biopsy, we commenced treatment with high-dose trimethoprim/sulfamethoxazole (5/25 mg/kg × 2/d), because toxoplasmosis was considered the most probable diagnosis. The metabarcoding analyses of brain tissue (but not CSF) yielded a 380-bp consensus sequence of the 18S rRNA gene with 100% similarity to a published *S. nesbitti* sequence (genomic DNA containing 18S rRNA gene; GenBank accession no. HF544323.1). On the basis of metabarcoding analyses and histological findings, we made a final diagnosis of neurosarcocystosis. *Sarcocystis* serologic testing was not obtainable.

The treatment regimen was well-tolerated, and the patient’s clinical and radiologic condition improved substantially without signs of immune reconstitution inflammatory syndrome; however, some neurologic sequelae remained. We tapered glucocorticoids gradually and started secondary prophylaxis of trimethoprim/sulfamethoxazole.

To our knowledge, human neurosarcocystosis is not recognized as an opportunistic infection. Given the phylogenetic relationship of *Sarcocystis* with *Toxoplasma*, the patient’s condition might represent reactivation of latent sarcocystis infection resulting from HIV-induced immunodeficiency. Because of limited knowledge about the dynamics of extraintestinal sarcocystosis in immunosuppressed hosts, we cannot determine whether this condition represents a primary infection or reactivation. A detailed travel history revealed no visits to *Sarcocystis*-endemic hotspots such as the Pangkor or Tioman Islands of Malaysia (*8*).

We hypothesize that *Sarcocystis* spp. may cause opportunistic CNS infections in immunocompromised persons. Furthermore, neurosarcocystosis might be misdiagnosed as toxoplasmosis clinically, histopathologically, and radiologically. Because both conditions respond to high-dose trimethoprim/sulfamethoxazole, therapeutic efficacy might inadvertently support such a misdiagnosis. This case illustrates that the true prevalence and disease patterns of opportunistic pathogens are probably underestimated and that routine microbiologic workup might fail to reveal rare and unrecognized opportunistic infections.
